# G-OnRamp: a Galaxy-based platform for collaborative annotation of eukaryotic genomes

**DOI:** 10.1093/bioinformatics/btz309

**Published:** 2019-05-09

**Authors:** Yating Liu, Luke Sargent, Wilson Leung, Sarah C R Elgin, Jeremy Goecks

**Affiliations:** 1 Department of Biology, Washington University in St. Louis, St. Louis, MO, USA; 2 Computational Biology Program, Oregon Health and Science University, Portland, OR, USA

## Abstract

**Summary:**

G-OnRamp provides a user-friendly, web-based platform for collaborative, end-to-end annotation of eukaryotic genomes using UCSC Assembly Hubs and JBrowse/Apollo genome browsers with evidence tracks derived from sequence alignments, *ab initio* gene predictors, RNA-Seq data and repeat finders. G-OnRamp can be used to visualize large genomics datasets and to perform collaborative genome annotation projects in both research and educational settings.

**Availability and implementation:**

The virtual machine images and tutorials are available on the G-OnRamp web site (http://g-onramp.org/deployments). The source code is available under an Academic Free License version 3.0 through the goeckslab GitHub repository (https://github.com/goeckslab).

**Supplementary information:**

Supplementary data are available at *Bioinformatics* online.

## 1 Introduction

Eukaryotic genomes are being sequenced at a rapid rate (Cheng *et al.*, 2018). The first task after genome sequencing is often annotating the genome with the locations of functional elements such as genes (exons, introns, splice sites) and promoters. High-quality genome annotations are critical for understanding organism function and evolution. Genome browsers facilitate annotation, enabling researchers to visually synthesize results from different experiments. Genome annotation requires the integration of multiple lines of evidence, both experimental (e.g. RNA-Seq) and computational (e.g. gene predictions, sequence alignments). However, generating evidence tracks and visualizations needed to analyze eukaryotic genomes remains technically challenging and time-consuming.

Many analysis workflows for genome annotation are already available (e.g. Hoff *et al.*, 2016; Holt and Yandell, 2011), but these are often difficult for biologists with limited bioinformatics expertise to use. Key challenges include configuring tools and software dependencies, learning to use command-line tools, running tools on a computing cluster, converting the results for visualization [e.g. using the UCSC Genome Browser (Kent *et al.*, 2002) or JBrowse (Buels *et al.*, 2016)], maintaining web servers with the visualization platform for collaboration with other researchers, and keeping track of analysis steps and parameters in order to re-use the workflow on other genome assemblies.

To address these challenges, we have developed G-OnRamp, a scalable user-friendly web-based platform for eukaryotic genome annotation as part of a collaboration between the Genomics Education Partnership (GEP; http://gep.wustl.edu) and Galaxy (https://galaxyproject.org). G-OnRamp has computational workflows that combine more than 25 community and custom analysis tools to create evidence tracks leading to complete UCSC Assembly Hubs (Raney *et al.*, 2014) and JBrowse/Apollo (Lee *et al.*, 2013) genome browsers that can be used for collaborative genome annotations. G-OnRamp output includes evidence tracks for homologous protein and transcript sequence alignments, *ab initio* gene predictions, transcriptional activity (full transcripts and splice junctions) and repeats. G-OnRamp can be deployed on the Amazon cloud platform via CloudLaunch (Afgan *et al.*, 2018) or locally via a virtual appliance. Genome browsers created with G-OnRamp can be exported to the CyVerse Data Store (Merchant *et al.*, 2016), where they can be used without needing a web server. G-OnRamp training materials and documentation are available at http://g-onramp.org/training (see Supplementary Text 1).

## 2 A complete platform for genome annotation

Galaxy is an open, web-based platform for accessible, reproducible and transparent analyses of large biological datasets that is used by thousands of scientists throughout the world (Afgan *et al.*, 2018). G-OnRamp extends Galaxy by providing the data analyses and conversions needed for constructing genome browsers for annotation.

G-OnRamp encapsulates the steps for constructing UCSC Assembly Hubs and JBrowse genome browsers using Galaxy workflows. Users specify input datasets—a genome assembly and RNA-Seq data from the target genome, transcript and protein sequences from a related informant genome—and then run the workflow to create the genome browser. G-OnRamp can be used to generate a genome browser for annotation of almost any eukaryotic genome. Supplementary Text 2 lists compute requirements for several genomes, with example data in Supplementary Text 3.

### 2.1 Sub-workflows to generate data for genome annotation

**Fig. 1.**
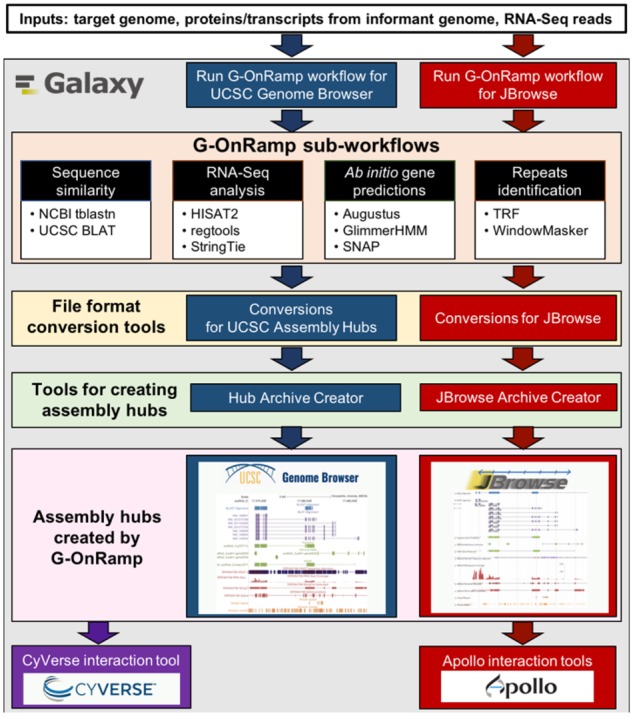
G-OnRamp overview. Inputs for the G-OnRamp workflow are the genome assembly and RNA-Seq data from the target organism, transcript and protein sequences from an informant genome. Sub-workflows that generate data for annotation are sequence similarity, RNA-Seq analysis, *ab initio* gene predictions and repeats identification. Data produced by the sub-workflows is used to create UCSC Assembly Hubs and JBrowse genome browsers. The Apollo interaction tools can convert the JBrowse genome browser into an Apollo instance for collaborative annotation. The CyVerse interaction tool can transfer the browsers to the CyVerse Data Store for long-term storage and visualization

The G-OnRamp workflow consists of four sub-workflows: homologous sequence similarity, RNA-Seq analysis, *ab initio* gene predictions and repeat identification (Fig. 1). Each sub-workflow is composed of multiple bioinformatics tools. See Supplementary Text 4 and the G-OnRamp web site (http://g-onramp.org) for details on the key components of each sub-workflow. The sub-workflows outputs provide the input data for the novel Galaxy tools, the Hub Archive Creator (HAC) and the JBrowse Archive Creator (JAC) to create genome browsers for the target genome. Supplementary Text 5 describes how to customize the workflow.

### 2.2 Visualization, collaboration and data storage tools

**Constructing Genome Browsers:** The HAC and JAC aggregate data produced by the sub-workflows to create UCSC Assembly Hubs and JBrowse genome browsers. Customization options include setting names and colors of each evidence track, grouping of evidence tracks and incorporation of custom evidence tracks into the genome browsers.

**Collaborative annotation with Apollo:** The ‘Create or Update Organism’ tool creates an Apollo instance from the JAC output. The ‘Apollo User Manager’ tool provides batch management by administrators of Apollo user accounts and roles, facilitating collaborative annotation. These G-OnRamp tools are based on tools developed by the Galaxy Genome Annotation project (https://github.com/galaxy-genome-annotation/galaxy-tools).

**Data storage and visualization:** G-OnRamp provides tools for creating an Apollo instance and for transferring G-OnRamp output to the CyVerse Data Store for long-term storage and visualization. Over 20 genome browsers that have been produced by G-OnRamp are available through the CyVerse Data Store at https://de.cyverse.org/anon-files/iplant/home/shared/G-OnRamp_hubs/index.html.

## 3 Conclusions

G-OnRamp is a scalable, user-friendly system for individual or collaborative eukaryotic genome annotation; it integrates the Galaxy platform, over 25 community and custom bioinformatics tools and the UCSC and JBrowse/Apollo genome browsers into a powerful annotation platform. G-OnRamp is useful in both research and educational settings.

## Funding

This work was supported by the National Institutes of Health [1R25 GM119157 to S.C.R.E.].

*Conflict of Interest*: none declared.

## Supplementary Material

btz309_Supplementary_Data
