# Supplementary material for: G-OnRamp: a Galaxy-based platform for collaborative annotation of eukaryotic genomes
Source: Bioinformatics. 2019 May 9;35(21):4422–3. doi: 10.1093/bioinformatics/btz309 (PMC6821377; doi:10.1093/bioinformatics/btz309)
Supplement: btz309_Supplementary_Data [file btz309_supplementary_data.zip › btz309-suppl_data/Supplement 1 - Table of training materials.docx]

**Supplement #1: Table of training materials**

The following curriculum materials are available through the “G-OnRamp Training Materials” page on the G-OnRamp web site (<http://g-onramp.org/training>):

| **#** | **Topic** | **Type** | **Duration** | **Difficulty** |
| --- | --- | --- | --- | --- |
| 1 | Overview of Galaxy | Lecture | 30m | Beginner |
| 2 | Using Galaxy to analyze RNA-Seq data | Walkthrough | 1hr | Beginner |
| 3 | Introduction to G-OnRamp | Lecture | 30m | Beginner |
| 4 | Introduction to G-OnRamp Walkthrough | Walkthrough | 1hr | Beginner |
| 5 | Customize the Genome Browsers produced by G-OnRamp | Walkthrough | 1hr30m | Advanced |
| 6 | Explore the Genome Browsers created by G-OnRamp | Walkthrough | 1hr30m | Advanced |
| 7 | Transfer large genomic datasets to G-OnRamp | Walkthrough | 1hr | Advanced |
| 8 | Use G-OnRamp to set up a collaborative annotation environment on Apollo | Lecture | 30m | Advanced |
| 9 | Set up a collaborative annotation environment on Apollo for Drosophila | Walkthrough | 1hr | Advanced |
| 10 | Use Apollo to do collaborative genome annotation | Walkthrough | 1hr | Advanced |
| 11 | Comparative Gene Annotation with Galaxy / G-OnRamp | Walkthrough | 1hr | Advanced |
| 12 | Deployment options for G-OnRamp (Keynote) | Lecture | 30m | Advanced |
| 13 | Deployment options for G-OnRamp (PDF) | Lecture | 30m | Advanced |
| 14 | Virtual Machine Installation Walkthrough | Walkthrough | 30m | Advanced |
| 15 | CloudLaunch Deployment Walkthrough | Walkthrough | 30m | Advanced |
| 16 | Storing & Accessing G-OnRamp’s Assembly Hubs outside of Galaxy | Lecture | 30m | Advanced |
| 17 | Differential expression analysis with RNA-Seq | Lecture | 1hr30m | Advanced |
| 18 | G-OnRamp Glossary | Supplement | 30m | Beginner |

**Links**

| **#** | **Title** | **Address** |
| --- | --- | --- |
| L1 | G-OnRamp Home Page | <http://g-onramp.org/> |
| L2 | Documentation & Training Materials | <http://g-onramp.org/training> |
| L3 | G-OnRamp Deployment Options | <http://g-onramp.org/deployments> |
| L4 | G-OnRamp Ansible Playbooks | <https://github.com/goeckslab/GOnRampKickStart> |

**Getting Started — Overview**

- Overview of Galaxy: Galaxy Community materials to become familiar with the interface, tools and conventions.
  - Reference materials: 1, 2
- Overview of G-OnRamp
  - Reference materials: L1, L2, 3
- G-OnRamp Glossary: Definitions for terms found in the G-OnRamp documentation and training materials
  - Reference material: 18

**Getting Started — Deployment Options**

- General overview of deployment options
  - Reference materials: L3, 12/13
- Launch the G-OnRamp server locally for training and small-scale testing
  - Quick, free exploration
  - Download the virtual appliance at <https://wustl.box.com/v/g-onramp-vm-v1>
  - Reference material: 14
- Launch the G-OnRamp server on the Cloud (Amazon EC2) with CloudLaunch
  - Production deployments for analysis of whole genome assemblies
  - Launch the virtual appliance at <https://launch.usegalaxy.org/> (incurs cost)
  - Reference material: 15
- Custom installations: Using Ansible to deploy to multiple platforms.
  - Reference materials: L4, 12/13

**Getting Started — Visualizing & Analyzing Data**

- Using G-OnRamp to create and explore genome browsers
  - Reference materials: 3, 4, 6
- Modify existing G-OnRamp workflows, add new tools to the workflow, add custom tracks to the genome browser
  - Reference material: 5

**Getting Started — Collaborative Genome Annotation with Apollo**

- Set up Apollo instance for collaborative annotation (create user accounts, workspaces)
  - Reference materials: 8, 9
- Comparative gene annotations using Apollo
  - Reference materials: 10, 11

**Getting Started — Data Transfer**

- Adding large data to G-OnRamp: use the built-in FTP server to efficiently transfer large datasets from remote sources
  - Reference material: 7
- Exporting genome browsers: store assembly hubs on the CyVerse data store for long-term storage and visualization
  - Reference material: 16

**Optional — Data Analysis Beyond Generating Genome Browsers and Gene Annotations**

- Differential expression analysis with RNA-Seq
  - Reference material: 17

**G-OnRamp Learning Workflow**

1. All skill levels: General reference and deployment materials
   - Getting G-OnRamp: 12/13, 14, 15
   - G-OnRamp Glossary: 18
2. Galaxy Novice: Unfamiliar with using Galaxy to analyze data
   - Galaxy 101/102 tutorials: 1, 2
3. G-OnRamp Novice: Familiar with Galaxy but have not used G-OnRamp
   - Using G-OnRamp to create a genome browser: 3, 4
   - Exploring a genome browser: 6
   - Using Apollo for gene annotations: 10, 11
4. Familiar with G-OnRamp: Advanced topics and customizations
   - Customize G-OnRamp workflows: 5
   - Customize Apollo: 8, 9
   - Transfer large datasets to G-OnRamp: 7
   - Export genome browser hubs to CyVerse: 16
   - Differential expression analysis with RNA-Seq: 17
