# Supplementary material for: G-OnRamp: a Galaxy-based platform for collaborative annotation of eukaryotic genomes
Source: Bioinformatics. 2019 May 9;35(21):4422–3. doi: 10.1093/bioinformatics/btz309 (PMC6821377; doi:10.1093/bioinformatics/btz309)
Supplement: btz309_Supplementary_Data [file btz309_supplementary_data.zip › btz309-suppl_data/Supplement 4 - Tools in each G-OnRamp sub-workflow.docx]

**Supplement #4: Tools in each G-OnRamp sub-workflow**

The key components of the four G-OnRamp sub-workflows are described below. Additional details are available on the “Learn G-OnRamp” page of the G-OnRamp web site (under the “Tools we use” section; <http://g-onramp.org/?page_id=32#tools>).

# Homologous sequence similarity

This sub-workflow uses NCBI tblastn (Gertz *et al.*, 2006; Camacho *et al.*, 2009) and BLAT (Kent, 2002) to align proteins and transcripts from an informant genome to the target genome. BLAT transcript alignments are filtered to determine the locations of the putative orthologs. The “UCSC Trix Index Generator” tool creates an index that enables users to search for protein and transcript matches by name.

# RNA-Seq analysis

This sub-workflow uses HISAT2 (Kim *et al.*, 2015) to align RNA-Seq reads to the target genome. The RNA-Seq read alignments are aggregated by the “Convert BAM to BigWig” tool to create a coverage track that shows the read alignment density. Putative splice junctions are catalogued by the “junctions extract” subprogram in regtools (<https://github.com/griffithlab/regtools>). Putative transcripts are assembled by StringTie (Pertea *et al.*, 2015) from the aligned RNA-Seq reads.

# *Ab initio* gene predictions

This sub-workflow uses the Augustus (Stanke *et al.*, 2006), GlimmerHMM (Majoros *et al.*, 2004), and SNAP (Korf, 2004) gene predictors to discover potential gene locations and sub-elements. Species-specific gene prediction parameters can be specified when the workflow is run.

# Repeats identification

Tandem Repeats Finder (Benson, 1999) and the TrfBig utility developed by the UCSC Genome Bioinformatics Group are used to identify tandem repeats. WindowMasker (Morgulis *et al.*, 2006) is used to identify transposon remnants and simple repeats.

# References

Benson,G. (1999) Tandem repeats finder: a program to analyze DNA sequences. *Nucleic Acids Res.*, **27**, 573–580.

Camacho,C. *et al.* (2009) BLAST+: architecture and applications. *BMC Bioinformatics*, **10**, 421.

Gertz,E.M. *et al.* (2006) Composition-based statistics and translated nucleotide searches: improving the TBLASTN module of BLAST. *BMC Biol.*, **4**, 41.

Kent,W.J. (2002) BLAT — the BLAST-like alignment tool. *Genome Res.*, **12**, 656–664.

Kim,D. *et al.* (2015) HISAT: a fast spliced aligner with low memory requirements. *Nat. Methods*, **12**, 357–360.

Korf,I. (2004) Gene finding in novel genomes. *BMC Bioinformatics*, **5**, 59.

Majoros,W.H. *et al.* (2004) TigrScan and GlimmerHMM: two open source ab initio eukaryotic gene-finders. *Bioinformatics*, **20**, 2878–2879.

Morgulis,A. *et al.* (2006) WindowMasker: window-based masker for sequenced genomes. *Bioinformatics*, **22**, 134–141.

Pertea,M. *et al.* (2015) StringTie enables improved reconstruction of a transcriptome from RNA-seq reads. *Nat. Biotechnol.*, **33**, 290–295.

Stanke,M. *et al.* (2006) Gene prediction in eukaryotes with a generalized hidden Markov model that uses hints from external sources. *BMC Bioinformatics*, **7**, 62.
