# Supplementary material for: G-OnRamp: a Galaxy-based platform for collaborative annotation of eukaryotic genomes
Source: Bioinformatics. 2019 May 9;35(21):4422–3. doi: 10.1093/bioinformatics/btz309 (PMC6821377; doi:10.1093/bioinformatics/btz309)
Supplement: btz309_Supplementary_Data [file btz309_supplementary_data.zip › btz309-suppl_data/Supplement 5 - Customize the G-OnRamp workflows.docx]

**Supplement #5: Customize the G-OnRamp workflows**

# Walkthrough on how to customize G-OnRamp workflows

The “[Customize the Genome Browsers produced by G-OnRamp](https://wustl.box.com/shared/static/9fpfn0vsu1stu5nu1bbanhdw4ucacxls.zip)” walkthrough describes how users can modify tool parameters, incorporate additional analysis tools (*e.g.*, WindowMasker) into the Galaxy workflow, and incorporate a custom track (*e.g.*, bigWig file) produced by other analysis tools to create a new UCSC Assembly Hub. This walkthrough is available on the “G-OnRamp Training Materials” page of the G-OnRamp web site (<http://g-onramp.org/training>).

# Strategies to incorporate additional evidence tracks into the Genome Browsers produced by G-OnRamp

The tools in the G-OnRamp workflows follow the standard conventions (*e.g.*, Datatypes, inputs, outputs) used by other tools in the Galaxy ecosystem (*e.g.*, tools in [Galaxy Main](https://usegalaxy.org/) and [Galaxy Tool Shed](https://toolshed.g2.bx.psu.edu/)). The Hub Archive Creator (HAC) and the JBrowse Archive Creator (JAC) accepts input data files in [standardized formats](https://galaxyproject.org/learn/datatypes/) (*e.g.*, BED, GFF3, GTF, BigWig, BAM). Consequently, users can use the Galaxy workflow Canvas to add new tools to the workflow directly, run the tools in a separate Galaxy workflow, or run the analysis outside of Galaxy and add the results as additional inputs to HAC and JAC in order to customize the evidence tracks available on the Genome Browsers produced by G-OnRamp.

For example, the [Gene Model Mapper](http://www.jstacs.de/index.php/GeMoMa) (GeMoMA) Galaxy workflow can be used to produce gene predictions that are based on sequence homology (Keilwagen *et al.*, 2018). The [mGene.web](http://www.mgene.org/) Galaxy workflow can be used to produce gene predictions that are based on support vector machines (Schweikert *et al.*, 2009). Similarly, an expert user can run gene prediction pipelines such as [MAKER](http://www.yandell-lab.org/software/maker.html) (Holt and Yandell, 2011) outside of Galaxy and then add the MAKER GFF3 output file as an additional input dataset to HAC and JAC.

# Challenges with incorporating additional tools into the G-OnRamp workflows

While the HAC and JAC accept data files in standard data formats, one of the challenges with the integration of additional tools into G-OnRamp is the conversion of output files produced by the bioinformatics analysis tools into file formats that are compatible with HAC and JAC. For example, some tools produce output files that are in older (deprecated) formats such as GFF2 ([exonerate](https://www.ebi.ac.uk/about/vertebrate-genomics/software/exonerate)), that do not conform to the format specification ([BRAKER](https://github.com/Gaius-Augustus/BRAKER)), or that are incompatible with downstream tools due to differences in the interpretation of the specifications (MAKER + [gff3ToGenePred](https://github.com/ucscGenomeBrowser/kent/tree/master/src/hg/utils/gff3ToGenePred)). Some tools also require specific program settings and the addition of specific attributes before the output files can be used with downstream tools (*e.g.*, [STAR](https://github.com/alexdobin/STAR) + [Cufflinks](https://github.com/cole-trapnell-lab/cufflinks)/[StringTie](https://ccb.jhu.edu/software/stringtie/), [minimap2](https://github.com/lh3/minimap2) + [StringTie](https://ccb.jhu.edu/software/stringtie/)).

Below are four examples which illustrates these challenges:

## 1. Output files that are in deprecated file formats

The [exonerate](https://www.ebi.ac.uk/about/vertebrate-genomics/software/exonerate) aligner generates output in GFF2 instead of GFF3 format. The GFF2 format has been [deprecated by GMOD](http://gmod.org/wiki/GFF2#The_GFF2_File_Format), and tools that convert files from GFF2 to GFF3 must make specific assumptions about the GFF2 data (see the “[Converting GFF2 to GFF3](http://gmod.org/wiki/GFF2#Converting_GFF2_to_GFF3)” section of the GMOD wiki page for details). In order to create the exonerate alignments in GFF3 format, one could use a fork of exonerate with GFF3 support (*e.g.*, [exonerate-gff3](https://github.com/hotdogee/exonerate-gff3)) or use custom conversion scripts (*e.g.*, [exonerate_gff_to_alignment_gff3.pl](https://github.com/EVidenceModeler/EVidenceModeler/blob/master/EvmUtils/misc/exonerate_gff_to_alignment_gff3.pl)) provided by tools such as EvidenceModeler (Haas *et al.*, 2008).

## 2. Output files that do not conform to the file format specification

Depending on the configuration settings, the [BRAKER](https://github.com/Gaius-Augustus/BRAKER) gene prediction pipeline (Hoff *et al.*, 2016) could produce non-conformant GFF3 files that lack the “exon” features. In that case, an additional data conversion step is needed to create the “exon” features based on the locations of the “CDS”, “five_prime_UTR”, and “three_prime_UTR” features (see <https://www.biostars.org/p/101225/>).

## 3. Compatibility issues due to different interpretation of the file format specification

The MAKER annotation workflow produces a GFF3 output file with attribute tags that begin with an underscore. For example the “_AED” tag corresponds to the Annotation Edit Distance and the “_QI” tag corresponds to the Quality Index (Campbell *et al.*, 2014). In order to display the gene predictions on the UCSC Assembly Hub, this GFF3 file must first be converted to the [bigGenePred](https://genome.ucsc.edu/goldenpath/help/bigGenePred.html) format. The UCSC Genome Bioinformatics Group provides the [gff3ToGenePred](https://github.com/ucscGenomeBrowser/kent/tree/master/src/hg/utils/gff3ToGenePred) tool to facilitate this data conversion. However, the tool requires all the attributes of the GFF3 feature to begin with an alphabetic character, even though this restriction is not required by the GFF3 specification (see <https://www.biostars.org/p/340948/>).

## 4. Compatibility issues due to program settings and attributes required by downstream tools

RNA-Seq alignment tools such as HISAT2 (Kim *et al.*, 2015) and STAR (Dobin *et al.*, 2013) must be run with specific settings in order for their alignment outputs to be compatible with transcriptome assembly tools such as StringTie (Pertea *et al.*, 2015) and Cufflinks (Trapnell *et al.*, 2012). For example, HISAT2 should be run with the “--downstream-transcriptome-assembly” option in order to optimize the alignment output for transcriptome assembly with StringTie. Similarly, HISAT2 should be run with the “--dta-cufflinks” option for the alignments to be optimized for transcriptome assembly with Cufflinks. (See the “Spliced alignment options” section of the [HISAT2 manual](https://ccb.jhu.edu/software/hisat2/manual.shtml) for details.)

For STAR, unstranded RNA-Seq data should be run with the “--outSAMstrandField intronMotif” option in order to add the XS strand attribute required by Cufflinks to the BAM alignment file. In contrast, stranded RNA-Seq data does not require any additional options for STAR, but it requires the “--library-type” option when the BAM file is analyzed by Cufflinks. (See the “Compatibility with Cufflinks/Cuffdiff” section of the [STAR manual](https://github.com/alexdobin/STAR/blob/master/doc/STARmanual.pdf) for details.)

StringTie has previously required the user to append the XS tag to the BAM alignment files produced by STAR (<https://github.com/gpertea/stringtie/issues/86>). The option to specify the stranded RNA-Seq library was added to StringTie in [release 1.3.2](http://ccb.jhu.edu/software/stringtie/index.shtml#news). The minimap2 aligner (Li, 2018) uses the “ts” tag instead of “XS” tag to denote the strand attribute (<https://github.com/lh3/minimap2/issues/28>). In previous releases of StringTie, these “ts” tags would need to be converted into the corresponding “XS” tags prior to transcriptome assembly. As of [version 1.3.5](http://ccb.jhu.edu/software/stringtie/index.shtml#news) (released on 11/2/2018), this modification is no longer needed as StringTie will treat the “ts” tag as an alternative to the “XS” tag. (Note that StringTie 1.3.5 is not yet available on the Galaxy Tool Shed as of 2/28/2019.)

# References

Campbell,M.S. *et al.* (2014) Genome Annotation and Curation Using MAKER and MAKER-P. *Curr Protoc Bioinformatics*, **48**, 4.11.1-39.

Dobin,A. *et al.* (2013) STAR: ultrafast universal RNA-seq aligner. *Bioinformatics*, **29**, 15–21.

Haas,B.J. *et al.* (2008) Automated eukaryotic gene structure annotation using EVidenceModeler and the Program to Assemble Spliced Alignments. *Genome Biol.*, **9**, R7.

Hoff,K.J. *et al.* (2016) BRAKER1: Unsupervised RNA-Seq-Based Genome Annotation with GeneMark-ET and AUGUSTUS. *Bioinformatics*, **32**, 767–769.

Holt,C. and Yandell,M. (2011) MAKER2: an annotation pipeline and genome-database management tool for second-generation genome projects. *BMC Bioinformatics*, **12**, 491.

Keilwagen,J. *et al.* (2018) Combining RNA-seq data and homology-based gene prediction for plants, animals and fungi. *BMC Bioinformatics*, **19**, 189.

Kim,D. *et al.* (2015) HISAT: a fast spliced aligner with low memory requirements. *Nat. Methods*, **12**, 357–360.

Li,H. (2018) Minimap2: pairwise alignment for nucleotide sequences. *Bioinformatics*, **34**, 3094–3100.

Pertea,M. *et al.* (2015) StringTie enables improved reconstruction of a transcriptome from RNA-seq reads. *Nat. Biotechnol.*, **33**, 290–295.

Schweikert,G. *et al.* (2009) mGene: accurate SVM-based gene finding with an application to nematode genomes. *Genome Res.*, **19**, 2133–2143.

Trapnell,C. *et al.* (2012) Differential gene and transcript expression analysis of RNA-seq experiments with TopHat and Cufflinks. *Nat Protoc*, **7**, 562–578.
